# Supplementary material for: Evaluation of follow-up colposcopy procedures after abnormal cervical screening result across a statewide study in Mississippi
Source: Cancer Causes Control. 2024 Aug 17;35(11):1487–96. doi: 10.1007/s10552-024-01905-0 (PMC11561063; doi:10.1007/s10552-024-01905-0)
Supplement: Supplementary file 1 — Supplementary file1 (DOCX 41 kb) [file 10552_2024_1905_MOESM1_ESM.docx]

**Supplemental Table S1**

Age-stratified HPV results by cytology category for Individuals Following Abnormal Cervical Cancer Screening

| Cytology | HPV 16/18 | | HPV Other HR12 | | HPV - | | HPV Missing | |
| --- | --- | --- | --- | --- | --- | --- | --- | --- |
|  | N | row % | N | row % | N | row% | N | row% |
| Age < 30 |  |  |  |  |  |  |  |  |
| ASC-US | 54 | 12.6 | 370 | 86.4 | 4 | 0.9 | -- | -- |
| Age ≥ 30 |  |  |  |  |  |  |  |  |
| Inadequate | 1 | 100 | -- | -- | -- | -- | -- | -- |
| NILM | 164 | 99.4 | 1 | 0.6 | -- | -- | -- | -- |
| ASC-US | 45 | 24.7 | 130 | 74.3 | -- | -- | -- | -- |
| LSIL | 29 | 26.4 | 77 | 70.0 | 2 | 1.8 | 2 | 1.8 |
| ASC-H | 5 | 21.7 | 10 | 43.5 | 8 | 34.8 | -- | -- |
| HSIL | 36 | 51.4 | 32 | 45.7 | 2 | 2.9 | -- | -- |
| Atyp/EM/glandular | 1 | 9.1 | 1 | 9.1 | 9 | 81.8 | -- | -- |

*Note.* Abbreviations: ASC-H, atypical squamous cells cannot exclude high grade; ASC-US, atypical squamous cells of undetermined significance; HSIL, high-grade intraepithelial lesion; HPV 16/18 = HPV 16, HPV 18; HPV Other HR12 = HPV 31, 33, 35, 39, 45, 51, 52, 56, 58, 59, 66, and 68; LSIL, low-grade intraepithelial lesion; NILM, negative for intraepithelial lesion or malignancy.

**Supplemental Table S2**

Age-stratified HPV positive results by cytology category for Individuals Following Abnormal Cervical Cancer Screening

| Cytology | Total | | Colpo | | No Colpo | | *P* Value |
| --- | --- | --- | --- | --- | --- | --- | --- |
|  | N | col % | N | row % | N | row % |  |
| Age < 30 Years |  |  |  |  |  |  |  |
| ASC-US |  |  |  |  |  |  | .387 |
| HPV 16/18 | 54 | 12.6 | 25 | 46.3 | 29 | 53.7 |  |
| HPV Other HR12 | 370 | 86.2 | 133 | 35.9 | 237 | 64.1 |  |
| Age ≥ 30 Years |  |  |  |  |  |  |  |
| Inadequate/NILM |  |  |  |  |  |  |  |
| HPV 16/18 | 165 | 100 | 91 | 55.2 | 74 | 44.8 |  |
| ASC-US |  |  |  |  |  |  | .670 |
| HPV 16/18 | 45 | 25.7 | 32 | 71.1 | 13 | 28.9 |  |
| HPV Other HR12 | 130 | 74.3 | 88 | 67.7 | 42 | 32.2 |  |
| LSIL |  |  |  |  |  |  | .573 |
| HPV 16/18 | 29 | 27.4 | 23 | 79.3 | 6 | 20.7 |  |
| HPV Other HR12 | 77 | 72.6 | 57 | 74.0 | 20 | 26.0 |  |
| ASC-H |  |  |  |  |  |  | .231* |
| HPV 16/18 | 5 | 33.3 | 5 | 100 | -- | -- |  |
| HPV Other HR12 | 10 | 66.7 | 6 | 60.0 | 4 | 40.0 |  |
| HSIL |  |  |  |  |  |  | .056 |
| HPV 16/18 | 36 | 52.9 | 23 | 63.9 | 13 | 36.1 |  |
| HPV Other HR12 | 32 | 47.1 | 27 | 84.4 | 5 | 15.6 |  |

*Note.* Abbreviations: ASC-H, atypical squamous cells cannot exclude high grade; ASC-US, atypical squamous cells of undetermined significance; HSIL, high-grade intraepithelial lesion; HPV 16/18 = HPV 16, HPV 18; HPV Other HR12 = HPV 31, 33, 35, 39, 45, 51, 52, 56, 58, 59, 66, and 68; LSIL, low-grade intraepithelial lesion; NILM, negative for intraepithelial lesion or malignancy.

P-values obtained from chi-square and fisher’s exact* analysis

A total of 1 HPV result on women <30 was missing. A total of 4 HPV results on women <30 were negative.

A total of 59 HPV results on women ≥ 30 were missing. A total of 21 HPV results on women ≥30 were negative.

Excludes one woman ≥ 30 with NILM HPV Other HR12 and two with endometrial/glandular cytology

**Supplemental Table S3**

Characteristics of those who came back for repeat Pap vs. no repeat Pap among those without Diagnostic follow-up

|  | Repeat Pap | | No Repeat Pap | | p-value |
| --- | --- | --- | --- | --- | --- |
|  | N | % | N | % |  |
| Age |  |  |  |  | .631 |
| <30 years old | 178 | 45.3 | 215 | 54.7 |  |
| ≥30 years old | 86 | 43.2 | 113 | 56.8 |  |
| Race |  |  |  |  | .312 |
| Non-Hispanic White | 67 | 46.5 | 77 | 53.5 |  |
| Non-Hispanic Black | 137 | 40.8 | 199 | 59.2 |  |
| All Hispanic | 10 | 47.6 | 11 | 52.4 |  |
| Other | 28 | 52.8 | 25 | 47.2 |  |
| BMI |  |  |  |  | .365 |
| <25 | 80 | 43.7 | 103 | 56.3 |  |
| 25-<30 | 65 | 45.8 | 77 | 54.2 |  |
| 30-<35 | 47 | 49.5 | 48 | 50.5 |  |
| 35+ | 52 | 38.2 | 84 | 61.8 |  |
| Smoking |  |  |  |  | .550 |
| Never Smoker | 156 | 46.4 | 180 | 53.6 |  |
| Former | 23 | 39.7 | 35 | 60.3 |  |
| Current | 85 | 43.1 | 112 | 56.9 |  |
| Cytology Diagnosis^a^ |  |  |  |  | .015 |
| Inadequate/NILM | 25 | 33.8 | 49 | 66.2 |  |
| ASC-US | 139 | 42.9 | 185 | 57.1 |  |
| LSIL | 62 | 48.4 | 66 | 51.6 |  |
| ASC-H | 14 | 77.8 | 4 | 22.2 |  |
| HSIL | 20 | 46.5 | 23 | 53.5 |  |
| Atypical | 4 | 80.0 | 1 | 20.0 |  |

*Note.* Abbreviations: ASC-H, atypical squamous cells cannot exclude high grade; ASC-US, atypical squamous cells of undetermined significance; HSIL, high-grade intraepithelial lesion; LSIL, low-grade intraepithelial lesion; NILM, negative for intraepithelial lesion or malignancy.

^a^Among <30 years, all but 2 ASC-US were HPV negative. Among ≥ 30 years, 6 tested HPV negative and 16 were missing HPV results.

P < .001
